# Proximal vs. Distal LAD Lesions in ST-Elevation Myocardial Infarction: Insights from ECG and Coronary Angiography

**DOI:** 10.3390/jcm14165637

**Published:** 2025-08-09

**Authors:** Marius Rus, Bianca Maria Negruțiu, Felicia Liana Andronie-Cioara, Georgeta Pasca, Claudia Teodora Judea Pusta, Cristian Sava, Adriana Ioana Ardelean, Mihaela-Simona Popoviciu, Claudia Elena Staniș

**Affiliations:** 1 Department of Medical Disciplines, Faculty of Medicine and Pharmacy, University of Oradea, 410073 Oradea, Romania; 2 Cardiology Department, Bihor Clinical Emergency Hospital, 410169 Oradea, Romania; 3 Department of Dental Medicine, Faculty of Medicine and Pharmacy, University of Oradea, 410073 Oradea, Romania; 4 Department of Psycho Neuroscience and Recovery, Faculty of Medicine and Pharmacy, University of Oradea, 410073 Oradea, Romania; 5 Department of Morphological Disciplines, Faculty of Medicine and Pharmacy, University of Oradea, 410073 Oradea, Romania; 6 Department of Preclinical Disciplines, Faculty of Medicine and Pharmacy, University of Oradea, 410073 Oradea, Romania; 7 Faculty of Medicine and Pharmacy, University of Oradea, 410073 Oradea, Romania

**Keywords:** ST-segment elevation myocardial infarction, left anterior descending artery, proximal LAD occlusion, ECG, coronary angiography, PCI, aVL, aVR

## Abstract

**Background:** The identification of the left anterior descending (LAD) artery as the culprit vessel in ST-segment elevation myocardial infarction (STEMI) is critical for rapid decision-making and targeted reperfusion. Electrocardiography (ECG) remains a vital diagnostic tool, especially in cases of no prior clinical or imaging data. This study evaluates the accuracy of 12-lead ECG in identifying LAD involvement and occlusion level, while examining the prognostic significance of proximal versus distal LAD lesions in the era of modern reperfusion techniques. **Methods:** Data from 382 patients with STEMI were analyzed, focusing on the correlation between specific ECG patterns, particularly ST-segment elevation in aVL and aVR, and coronary angiographic findings. The predictive performance of ECG in localizing proximal LAD lesions was assessed through sensitivity, specificity, and predictive values. Clinical outcomes at 30 days and 2.5 years were compared between patients with proximal and distal LAD occlusions. **Results:** ST-segment elevation ≥ 0.5 mm in aVL or elevation in aVR, when associated with elevation in at least two contiguous precordial leads (V2–V4), demonstrated good sensitivity and predictive value for proximal LAD occlusion. Contrary to earlier studies, no significant difference in short- or long-term clinical outcomes was observed between proximal and distal LAD occlusion groups, possibly reflecting improvements in percutaneous coronary intervention (PCI) techniques and modern pharmacotherapy. **Conclusions:** The 12-lead ECG remains a valuable tool for identifying LAD as the culprit artery and approximating lesion location. However, in the era of advanced reperfusion therapy, the prognostic value of proximal LAD occlusion may be less pronounced than previously thought. These findings support a nuanced interpretation of ECG in guiding acute management without overestimating the long-term prognostic weight of lesion location alone.

## 1. Introduction

Acute myocardial infarction (AMI), particularly anterior ST-segment elevation myocardial infarction (STEMI), is a critical cardiovascular emergency with significant prognostic implications. The left anterior descending (LAD) artery is the most frequent culprit vessel in anterior AMI due to its perfusion of a large portion of the left ventricular myocardium [1]. Accurate identification of LAD lesions and, more importantly, the location of stenosis are important for timely and successful treatment [1].

The 12-lead electrocardiogram (ECG) remains the most important tool in the early assessment of STEMI. It provides confirmation of transmural ischemia and also important clues about the infarct-related artery and potential location of the obstruction [2]. ST-segment elevation in specific leads, particularly aVL and aVR, has been associated with proximal LAD occlusion, with varying degrees of sensitivity and specificity reported in the literature [3,4]. However, the diagnostic performance of these markers can be influenced by anatomic variations, the presence of multivessel disease, and the timing of coronary angiography [5].

While previous studies in the literature have emphasized the poor prognosis associated with proximal LAD stenosis, more recent studies have questioned this association in the modern era of reperfusion therapy. Some studies suggest that, with contemporary management, including timely percutaneous coronary intervention (PCI) and optimized antithrombotic therapy, the difference in clinical outcomes between proximal and distal LAD lesions may be diminished [6]. For example, the CADILLAC trial demonstrated no significant statistical difference in one-year survival between proximal and distal LAD lesions when treated with PCI [7].

This study investigated the utility of ECG in identifying the LAD as the culprit vessel and determining the level of stenosis in patients with STEMI. Moreover, it examined whether proximal LAD obstruction presented a worse prognosis in the modern therapeutic era. By integrating angiographic findings and ECG analysis, this research aimed to refine clinical strategies for early decision-making in the management of AMI.

## 2. Materials and Methods

The study was conducted between 5 January 2022 and 5 June 2024, and the patients were selected from Bihor County Clinical Emergency Hospital. A total of 382 patients were selected and included in this study.

The inclusion criteria were as follows:

- Chest pain over 30 min;
- Symptomatology debut of maximum 12 h before hospital presentation;
- A 4 mm or higher summed ST-elevation on the ECG, measured from the J point (over 2 mm in at least two leads from DI, aVL, V1–V6 or at least 1 mm elevation in all 4 leads DII, DIII, V5–V6 or over 2 mm in at least 2 leads DII, DIII, V5–V6).

STEMI patients underwent primary percutaneous transluminal coronary angioplasty or fibrinolysis, based on individual characteristics and guidelines recommendations.

The exclusion criteria were as follows:

- Fibrinolysis contraindication;
- Sepsis;
- Aortic aneurysm with mobile thrombus;
- Absence of femoral pulse or bilateral femoral vascular graft;
- Coronary artery bypass;
- Severe acute renal failure (creatinine over 250 µmol/L);
- Diabetics under metformin treatment in the last 48 h;
- Over 3 h interval from presentation to catheterization;
- Acute coronary syndrome (ACS) (with Q wave or not) in the last 30 days;
- High risk during ambulance transportation (cardiogenic shock or heart failure with severe hypotension–systolic arterial pressure over 65 mmHg);
- Severe cardiac arrhythmias;
- The existence of left bundle branch block (LBBB) or left ventricular hypertrophy (LVH) on the ECG;
- The existence of right bundle branch block (RBBB) with left anterior fascicular block (LAFB) on the ECG;
- Cardiac pacemaker.

Our hypothesis was that occlusion of the LAD extended to the diagonal artery may cause ischemia of two opposite electrical parts, the antero-lateral and the inferior. This may further attenuate ST-segment elevation in aVL, which would result in an isoelectric ST-segment, or even a depressed ST-segment, despite a proximal LAD lesion.

The final end-point of the study was a combination between mortality, reinfarction, and stroke, occurring during a median 2.5-year period from the total duration of the study. Clinical reinfarction is a myocardial infarction following a previous myocardial infarction and whose cause was not PCI or coronary artery bypass grafting. Patients were entered into the database immediately after randomization.

Variables:

- ST-segment and T wave measurement method

The ST-segment was measured at the J-point, the TP segment representing the isoelectric line. We considered the T wave positive or negative if it was positioned 0.05 mm above or below the isoelectric line, with the measurement taking place 120 ms after the J point.

- Q wave

Pathologic Q waves were considered as Q waves presenting the following characteristics:

- Any Q wave duration ≥ 30 ms in leads V1–V3;
- Q wave height ≥ 1 mm and duration ≥ 30 ms in leads DI, DII, aVL, aVF, V4–V6 in ≥2 adjacent leads;
- R-wave duration over 40 ms and R/S ratio over 1 (in the absence of pre-excitation syndrome, right ventricular hypertrophy, or RBBB in leads V1–V2);
- Localization of myocardial infarction.

Anterior myocardial infarction was determined by ST-segment elevation ≥ 2 mm in at least two adjacent precordial leads V2–V4. Inferior myocardial infarction was determined by an ST-segment elevation ≥1 mm in ≥2 leads (DII, DIII, and aVF). In case of concomitant ST-segment elevation in leads V1–V3, the patients were included in the inferior MI group. If the sum of ST-segment elevations was greater in V1–V2 than in V2–V3, the patients were not included. Patients with inferior ST-segment elevation (in ≥2 leads) with concomitant ST-segment elevation in V4–V6, but not in V1–V3, were not included. Lateral infarctions were not analyzed in a separate group.

### 2.1. The Culprit Artery and Occlusion Position

The LAD was the culprit artery in patients with a maximum ST-segment elevation over 2 mm in at least two adjacent V2–V4 leads. In analyzing occlusion positioning with respect to side branches, we excluded patients who had clinically established myocardial infarction.

Three ECG patterns were compared to determine the lesion level of the LAD based on 12-lead ECG. A lesion in the proximal segment of the LAD was determined by

**Figure 1:**
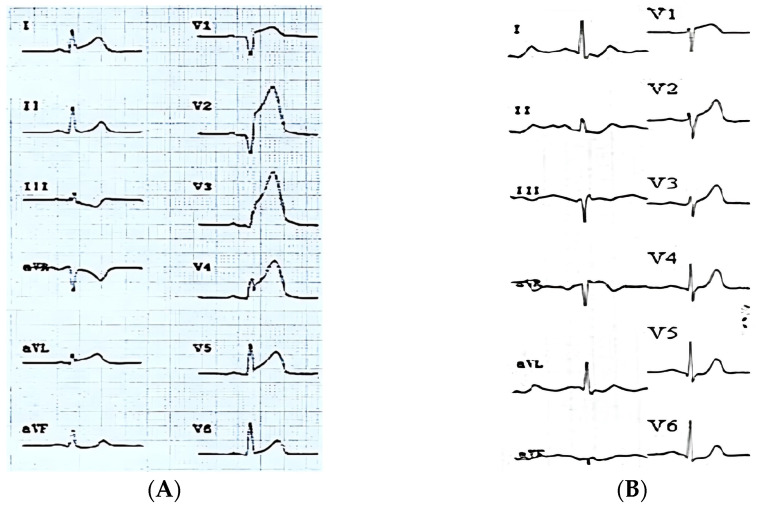
The maximum ST-segment elevation in leads V2–V3 in both ECGs indicates that the LAD is responsible for the infarction. (**A**) ST-segment elevation ≥ 0.5 mm in aVL and ST-segment depression in DIII, as reciprocal change, is a sign of proximal injury to the first diagonal branch, aVL + ECG pattern. (**B**) ST-segment elevation in aVR + ECG pattern.

**Figure 2:**
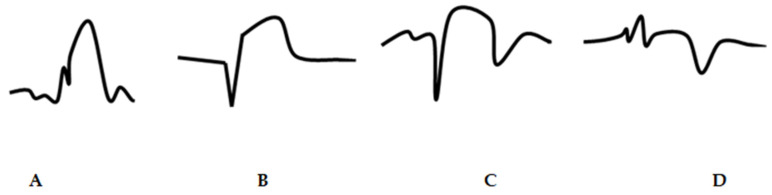
Different electrocardiogram patterns of evolving myocardial infarction and clinically established myocardial infarction in lead V3. (**A**) Evolving myocardial infarction: an ST-segment elevation and a peaked T wave without a Q wave. (**B**) Established clinical myocardial infarction without signs of reperfusion: a deep Q wave, an ST-segment elevation, and a positive T wave. (**C**) Established clinical myocardial infarction with incomplete reperfusion: ST-elevation and a biphasic T wave (negative terminal portion). (**D**) Clinically established myocardial infarction with complete reperfusion: minor ST-elevation and a negative T wave.

(1) Concomitant ST-segment elevation over 0.5 mm in lead aVL (aVL+ pattern) (Figure 1A);
(2) ST-segment elevation over 0.5 mm in aVL or any ST-segment elevation in lead aVR (pattern + aVR) (Figure 1B).

In concordance with our hypothesis, we defined proximal LAD lesion as pattern + aVR, excluding patients (*n* = 2) who presented simultaneous ST-segment elevation ≥ 1 mm in all inferior leads (proximal pattern). All the other ECG morphologies were categorized as distal occlusions. Data found from ECGs were correlated with coronary angiography performed in the acute phase.

### 2.2. ECG Definitions of Evolving Myocardial Infarction and Clinically Established Myocardial Infarction

Evolving myocardial infarction (eMI) can be defined as ST-segment elevation that fulfills the above criteria for anterior or inferior myocardial infarction, but no pathologic Q waves or inverted T waves are identified in leads with ST-segment elevation (Figure 2A) [8]. Clinically established myocardial infarction (cMI) can be defined by the appearance of pathologic Q waves and/or biphasic or negative T waves [8]. These ECG changes often represent myocardial necrosis or reperfusion [8]. It is agreed that negative T wave, as a term, is also used for patients with biphasic T wave with a negative terminal segment ≥ 0.05 mm [8]. Absence of reperfusion was defined as ST-elevation without T wave inversion (Figure 2B) [8]. Incomplete reperfusion was defined as ST-elevation with T wave inversion (Figure 2C) [8]. Complete reperfusion was defined as an isoelectric or small ST-segment with a completely inverted T wave (Figure 2D) [8].

### 2.3. Coronary Angiography

Coronary angiography was used to determine the culprit artery, as well as the localization and severity of luminal stenosis. The degree of stenosis of coronary arteries was assessed visually. A decrease in luminal diameter of >50% of a coronary vessel or side branch was considered significant.

When the artery presented more than one lesion, the culprit lesion was determined by identifying complete obstruction or detailed angiographic features of the lesion, including either the presence of residual thrombus or ulcerated plaque. The culprit lesion was considered proximal when it was located before a sizable (≥1.5 mm) diagonal branch.

### 2.4. Statistical Analysis

Categorical variables were expressed as number of patients or percentages and continuous variables as medians followed by IQ range. Statistical significance was determined using a chi-squared test or Fischer’s exact test for categorical variables and the Mann–Whitney test for numerical variables.

Susceptible, specific, positive, and negative values were calculated. The value of the confidence coefficient was set at 95% significance level. The rate of events was presented on a sample of 100 people correlated with the safety interval. The relative risk was analyzed by the Mantel–Haenszel method. Composite values of the data collected from subgroup ECGs were displayed as Kaplan–Meier curves. Comparisons between groups were made using the Log-Rank1 statistic. Cox regression analysis was employed to test the prognostic value of baseline characteristics and ECG variables in relation to the composite outcomes. The hazard ratio was also calculated, and the multivariate model included the following factors: age over 75 years, Killip class, heart rate > 100 beats/min, previous infarction type and location, more than 4 h to treatment, pathological history (diabetes or hypertension, smoking/non-smoking status, sex, use of lipid-lowering medication, aspirin use), ECG pattern, and treatment group. The effect of ECG pattern on composite outcomes was analyzed by evaluating the interaction with the treatment group (ECG pattern treatment group).

The Kappa coefficient (κ) represented the agreement between categorical values (e.g., ST-segment elevation ≥ 2 mm).

The Bland–Altman statistical method was used to determine agreement of variability in the interpretation of coronary angiograms using two different electrocardiographic measurement methods.

All the study data was analyzed with IBM SPSS Statistics 25 (IBM Corporation, Armonk, NY, USA), while illustrations were created using Microsoft Office Excel and Word 2024.

## 3. Results

Lateral STEMI rarely presented with massive ST-segment elevations (over 2 mm in at least two of the lateral leads (DI, aVL, V5–V6)). Patients whose ECG showed signs of ST-segment elevation supra-acute myocardial infarction were not included in the study, due to the small number of such cases (*n* = 2).

**Figure 3:**
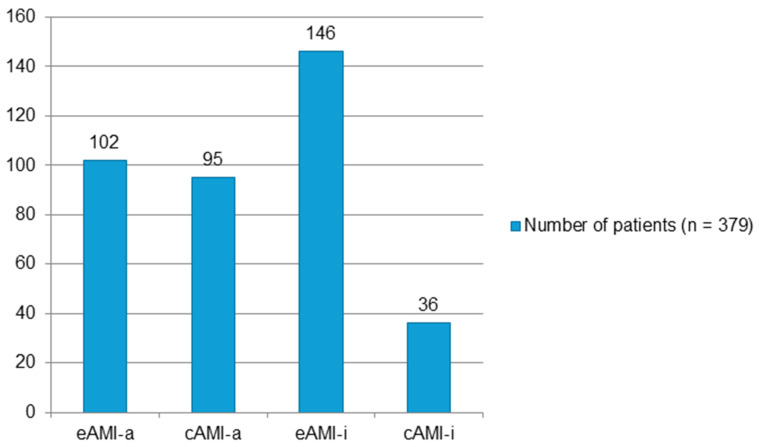
Distribution of patients with acute myocardial infarction according to ECG patterns and location. eAMI-a, evolving acute anterior myocardial infarction; cAMI-a, clinically established acute anterior myocardial infarction; eAMI-i, evolving acute inferior myocardial infarction; and cAMI-i, clinically established acute inferior myocardial infarction.

Of the 379 patients, 197 (51.98%) presented acute anterior myocardial infarction with ST-segment elevation, and 182 (48.02%) presented acute inferior myocardial infarction with ST-segment elevation, as shown by the ECG recordings. Of the patients with anterior STEMI, 102 had evolving AMI (eAMI-a), and 95 patients were diagnosed with clinically established AMI (cAMI-a) previously. Of the patients with ST-segment elevation of the inferior wall, 146 were diagnosed with evolving inferior AMI (eAMI-i), and 36 were diagnosed with clinically established inferior AMI (cAMI-i) (Figure 3).

The ECG pattern of eAMI was more prevalent in patients with inferior STEMI than in participants with anterior STEMI (*n* = 146, 59% vs. *n* = 102, 41%). In inferior STEMI, the ECG patterns of cAMI were more rare (27.5%).

**Figure 4:**
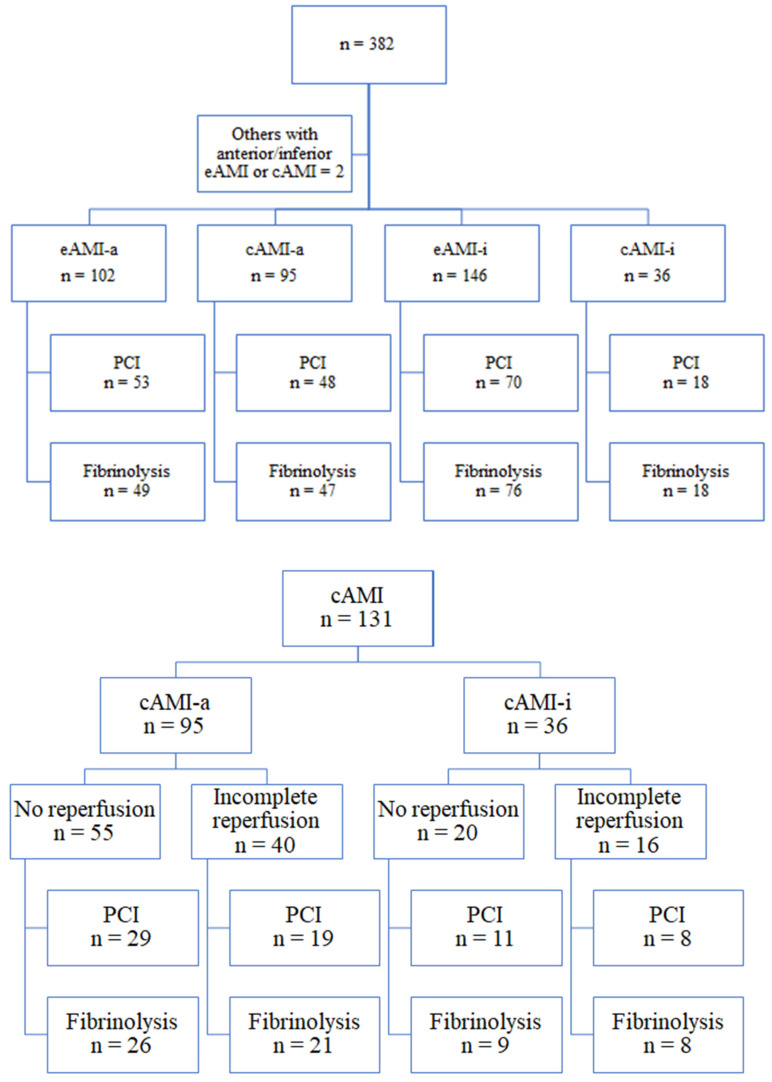
The subgroups of patients defined by electrocardiogram pattern in all patients (**top graph**) and clinically established myocardial infarction within the group (**bottom graph**). eAMI, evolving myocardial infarction; cAMI, clinically established myocardial infarction; and PCI, percutaneous coronary intervention.

Patients were divided into subgroups according to their ECGs: eAMI (*n* = 248; 65.43%) and cAMI (*n* = 131; 34.56%). The group with clinically established myocardial infarction was additionally observed according to signs of reperfusion. Figure 4 illustrates the ECG pattern of the subgroups of patients and their treatment.

### 3.1. Coronary Angiography Correspondence

Table 1 presents the distribution of angiographic findings, grouped by different ECG patterns. Only 6% of patients with previous anterior myocardial infarction presented non-LAD culprit lesions, whereas inferior STEMI occurred most frequently due to either RCA (right coronary artery) occlusions (74%) or LCX (left circumflex artery) occlusions (15%).

### 3.2. LAD Occlusion as Culprit Lesion

**Figure 5:**
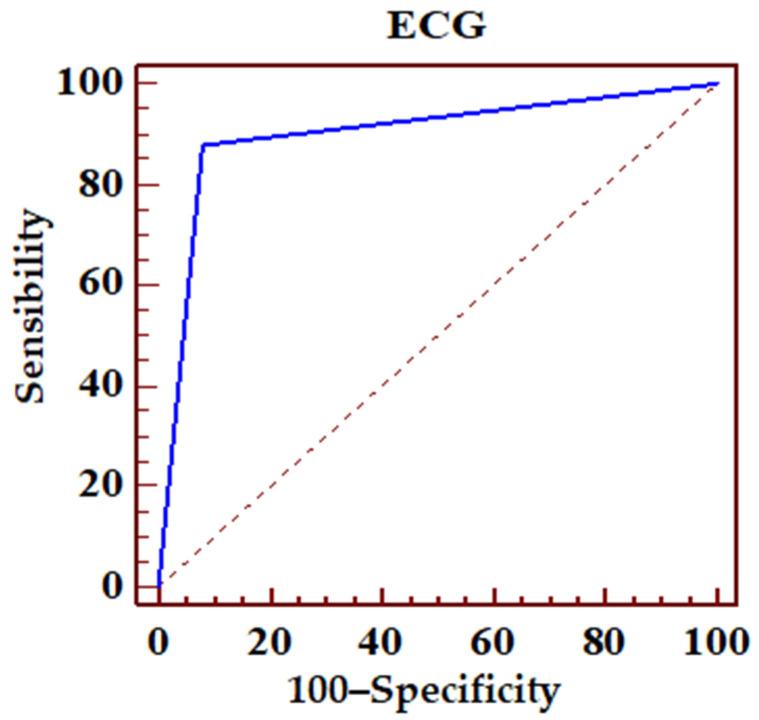
ECG sensibility and specificity in identifying LAD occlusion. Dotted line, random classifier.

The preestablished ECG criteria for LAD occlusion (*n* = 97), predicting the angiography results, presented 87.8% sensitivity and 92% specificity. The positive predictive values (PPV) and negative predictive values (NPV) were also high, 82.8% and 94.5%, respectively (Figure 5; Table 2).

**Figure 6:**
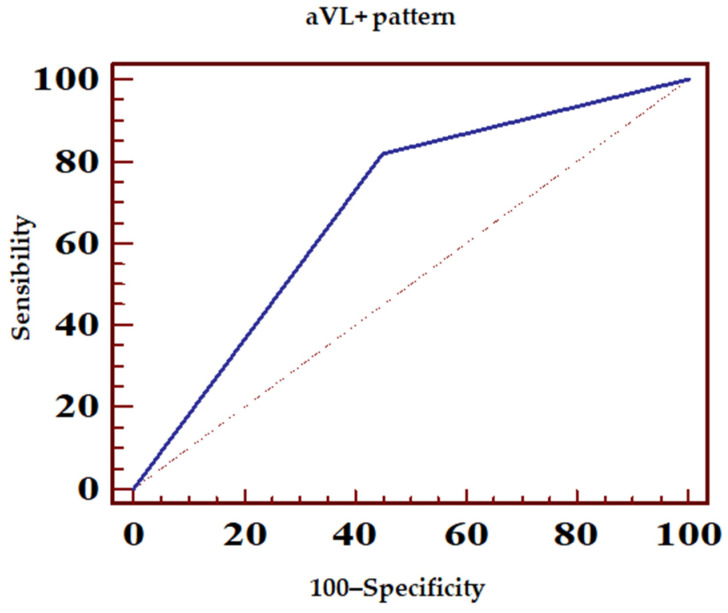
ECG sensibility and specificity of aVL+ pattern in predicting LAD occlusion. Dotted line, random classifier.

**Figure 7:**
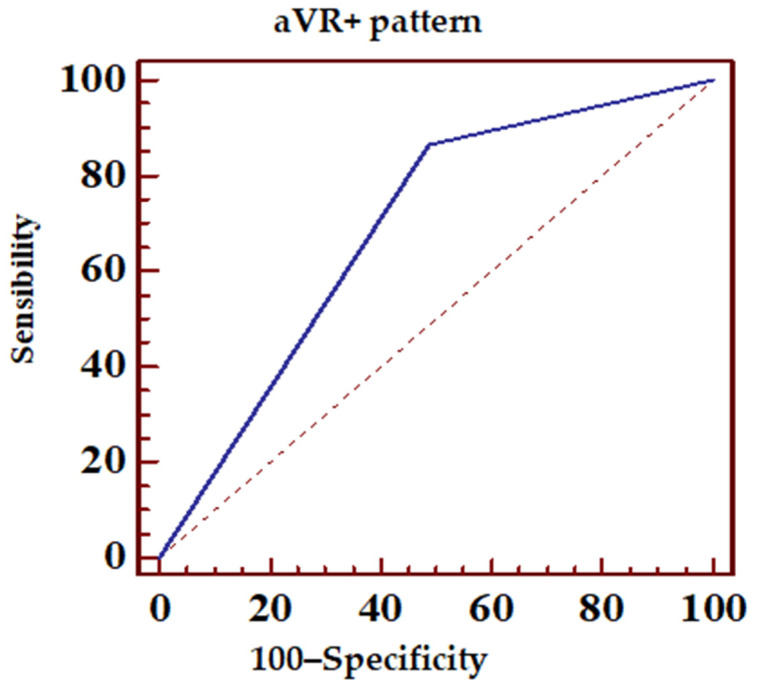
ECG sensibility and specificity of aVR+ pattern in predicting LAD occlusion. Dotted line, random classifier.

**Figure 8:**
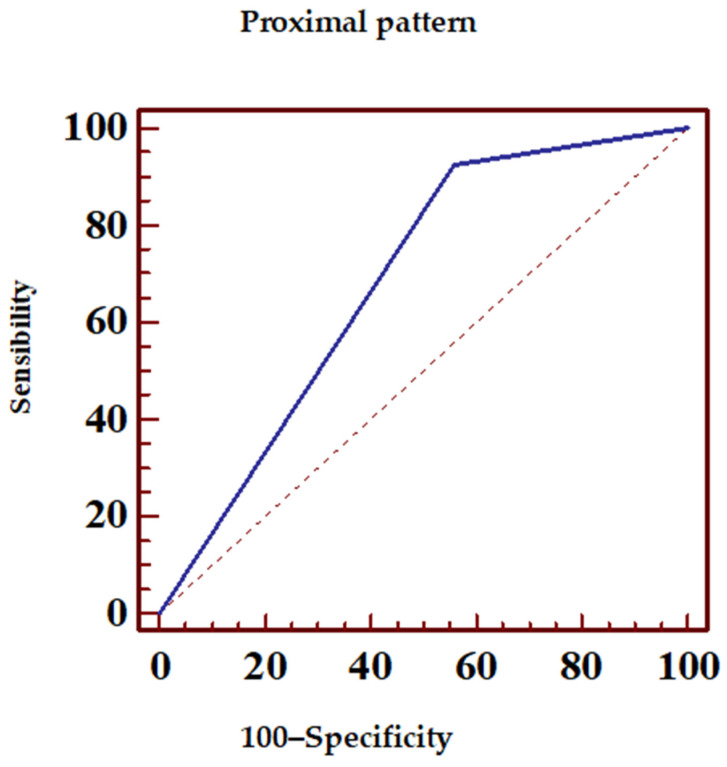
ECG sensibility and specificity of the proximal pattern in predicting LAD occlusion. Dotted line, random classifier.

Table 3 presents the sensitivity, specificity, and predictive values of various ECG patterns (N = 98) in predicting the location of LAD occlusion in relation to the lateral diagonal branches. The following patterns were described:

▪ aVL+ with ST-elevation ≥ 0.5 mm in the aVL lead (Figure 6, Table 4);
▪ aVR+ or aVL+ or any ST-elevation in the aVR lead (Figure 7, Table 5);
▪ Proximal pattern, aVR+ excluding pattern with ≥ 1 mm ST-elevation in all inferior leads (Figure 8, Table 6).

LAD proximal occlusion could be determined with 92.5% sensitivity and specificity of 44.1% with the proximal preestablished pattern. This pattern proves the existence of positive (83.8%) and negative (65.2%) predictive values regarding the anticipation of the proximal LAD occlusion found during coronary angiography.

**Figure 9:**
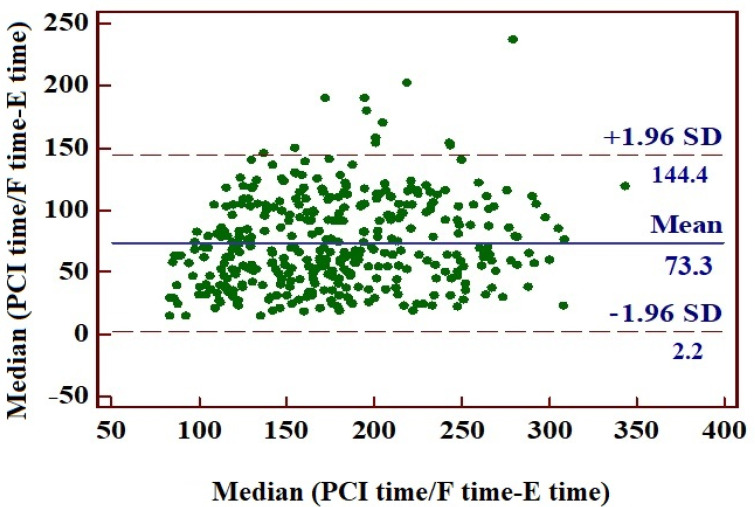
Remainder between time elapsed from the first appearance of symptoms to ECG recording and the time to PCI or fibrinolysis.

**Figure 10:**
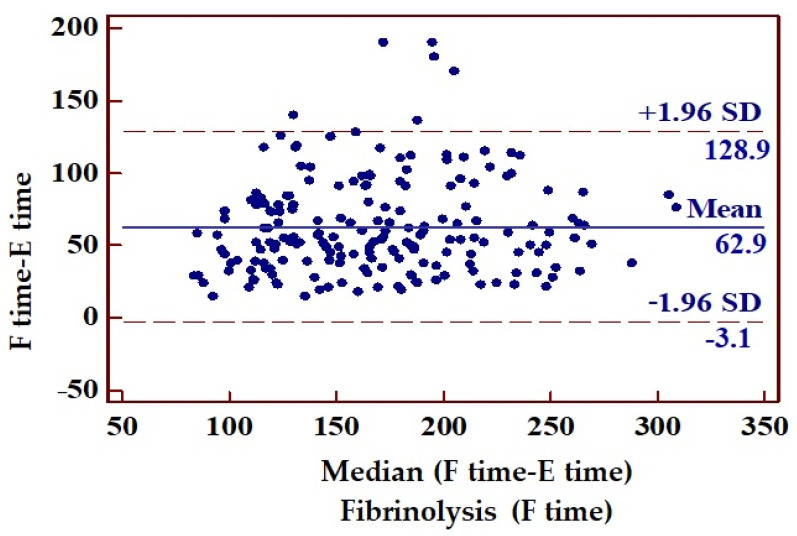
Remainder between time elapsed from the first appearance of symptoms to ECG recording and the time to fibrinolysis.

**Figure 11:**
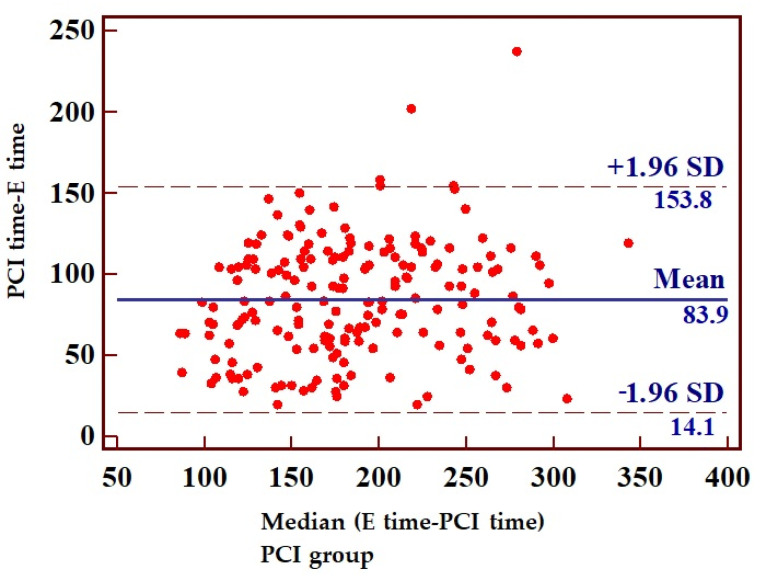
Remainder between time elapsed from the first appearance of symptoms ECG recording and the time to PCI.

The mean interval from symptom onset to ECG recording was 2 h and 21 min (95% confidence interval: 135.89–147.54 min), while the mean time to percutaneous coronary intervention (first balloon inflation) or fibrinolysis was 3 h and 35 min (193.07–205.99 min). Consequently, the mean delay from ECG recording to PCI or fibrinolysis was 73 min. Looking at the subgroups of patients who received PCI or fibrinolysis, there was a longer delay to PCI compared with the fibrinolysis group (*p* < 0.0001) (Figure 9, Figure 10 and Figure 11, Table 7).

### 3.3. Proximal LAD Occlusion—Effects on the Results

**Figure 12:**
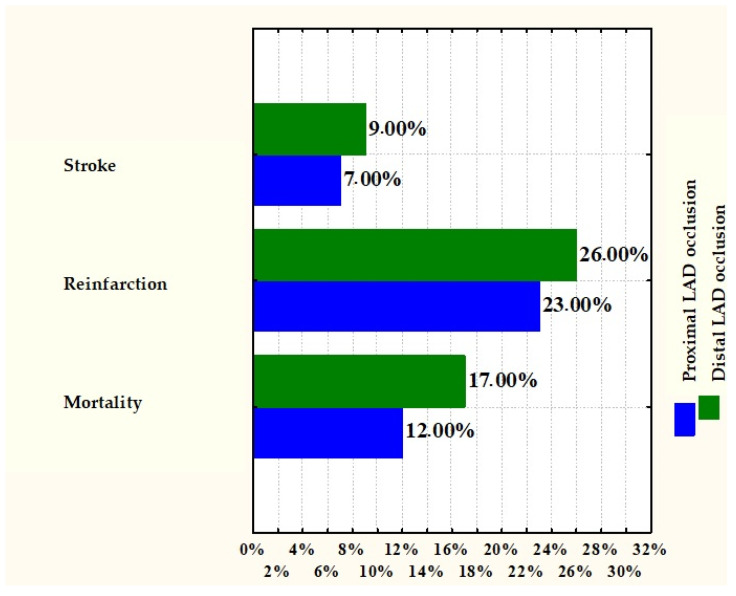
Stroke, reinfarction, and mortality in patients with proximal and distal LAD occlusion.

**Figure 13:**
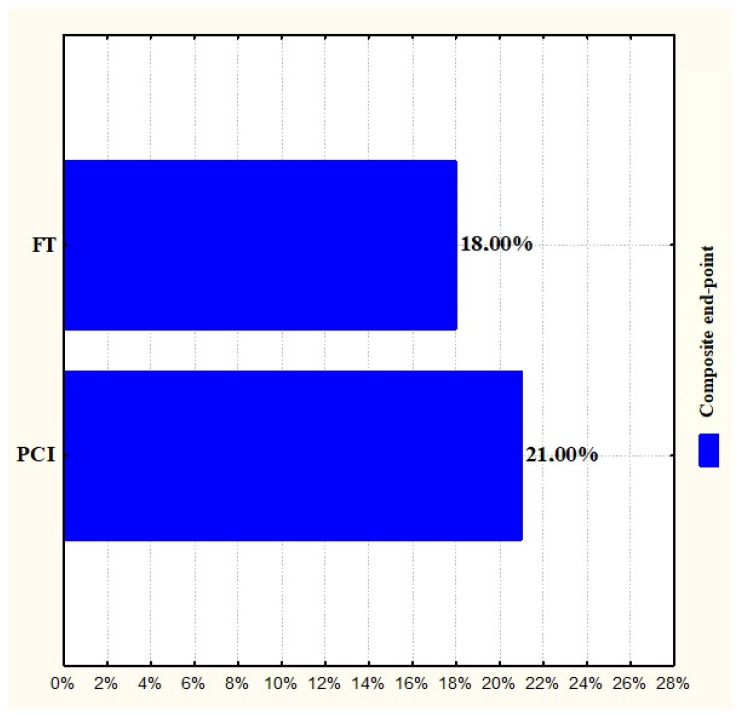
Final end-points in patients with proximal LAD occlusion (comparison between PCI and FT). PCI, percutaneous coronary intervention; FT, fibrinolysis time.

There were no significant differences in mortality, reinfarction, and stroke at 30 days and 2.5 years between proximal and distal angiographic lesions (12 vs. 17%, *p* = 0.38, 23 vs. 26%, *p* = 0.71, 7 vs. 9%, *p* = 0.76) (Figure 12; Table 8). At the 2.5-year mark, the incidence of the composite end-point was similar between the PCI and fibrinolysis groups in patients with ECG-documented proximal LAD occlusion (21% vs. 18%, *p* = 0.68; Figure 13).

## 4. Discussions

### 4.1. LAD—The Culprit Artery in AMI

The results present the information provided by the 12-lead ECG analysis without knowledge of the patient’s cardiovascular history, such as cardiomyopathies, previous myocardial infarction, previous ECGs, or other clinical patient data.

LAD lesion was detected with 87.8% sensitivity and 92% specificity in a broad spectrum of STEMI patients. Both negative and positive predictive values were also high, 94.5% and 82.8%, respectively. Thus, the ECG represented an important tool in identifying LAD as the culprit vessel, even when the patterns could be confusing [2].

### 4.2. LAD Occlusion Location

A relatively small number of studies have been published with reference to the comparison of different ECG patterns with coronary anatomy to determine the culprit vessels and lesion level [9,10,11]. Although multivessel disease is commonly encountered in clinical practice, most of these studies excluded such patients [12]. Moreover, there is the issue of time to angiography. Thus, the “gold standard” of coronary angiography, was performed within up to 2 weeks after fibrinolytic therapy in most cases included in studies.

The site of coronary obstruction does not always correspond to the extent or location of the resulting ischemia. In most cases, a plaque dissection is created at the site of the bifurcation. The thrombus may evolve proximally or distally, occluding one or numerous side branches during acute ischemia or later. After clot dissolution, complete or partial, the side branches may appear patent. Estimating if the thrombotic process has extended proximally during the acute phase (e.g., covering part of the side branch) can be challenging when PCI is performed later in the disease course. Furthermore, coronary angiography does not provide data on the collateral circulation that might accomplish perfusion of the myocardial segments in the territory of the artery responsible for the infarction. Some works suggest that even visible collateral vessels may disappear shortly after recanalization of the culprit artery [13]. Optimally, similar to the present study, the ECG pattern and coronary angiography images should be recorded as early as possible in the acute phase of myocardial infarction [14].

In the context of proximal LAD lesions, ST-segment elevation in lead aVL (and frequently in lead DI) has been associated with involvement of the first diagonal branch, which perfuses the antero-lateral myocardial wall and is typically occluded as a result of the thrombotic process [15,16,17]. The lesion vector in antero-lateral myocardial infarction is thus directed toward the left shoulder, toward the DI and aVL leads. The present study demonstrated a positive predictive value (PPV) of 78% and a negative predictive value (NPV) of 61.1% for ST-segment elevation ≥ 0.5 mm in lead aVL as an indicator of a proximal LAD lesion, findings that closely align with those reported by Arbane and Goy in a prospective study (PPV 81%, NPV 57%) in which coronary angiography was similarly performed within hours of symptom onset [17]. However, the sensitivity (82.05%) and specificity (55%) observed in the current analysis differ from those reported in the aforementioned study, which documented a lower sensitivity of 58% and a higher specificity of 81%.

In the study by Arbane and Goy [17], ST-segment elevation was measured at 80 ms after the J point in lead aVL, whereas the present study used a criterion of 0.5 mm elevation at the J point itself. The variation in ST-segment measurement criteria may explain the higher specificity reported by Arbane and Goy, compared to the greater sensitivity observed in the current analysis.

When there is no opposing lesion vector in the inferior segment, a greater elevation is present in the antero-lateral ECG leads. This could explain how ST-elevation ≥ 1 mm in the aVL lead is a specific but elusive indicator of proximal LAD lesion. Kim et al. [18] reported a high sensitivity of 91% and a specificity of 90% of the ST-segment pattern in lead aVL in one of the following situations:

1. ST-elevation ≥ 0.5 mm;
2. A smaller ST-segment elevation with T wave inversion;
3. Isoelectric ST-segment symmetrically associated with T wave inversion and atypical Q wave when AMI was a consequence of obstruction proximal to the first diagonal branch.

The aforementioned study included patients with advanced clinically established myocardial infarction. Another limitation was the time to coronary angiography, which was 6.3 days on average after the initial ECG. The study by Koju et al. [19], while excluding patients with organic heart disease and previous anterior myocardial infarction with ST-segment elevation ≥ 0.05 mm in aVL or aVR, identified a proximal LAD lesion with 73% and 42% sensitivity, as well as specificity of 78% and 97%, respectively. Coronary angiography was conducted within two weeks after the acute phase.

In the present study, it was observed that the presence of ST-segment elevation in the aVR lead, irrespective of changes occurring in other leads, improved the ECG sensitivity in predicting LAD lesion compared to ST-segment elevation in the aVL lead alone (sensitivity 86.42% vs. specificity 51.35%).

Engelen and colleagues [16] demonstrated that ST-segment elevation in aVR was a specific indicator of proximal LAD lesion, occurring before the first septal branch. The authors hypothesized that transmural ischemia of the basal septum, producing an electrical vector directed toward the right shoulder, could explain the ECG findings. On the other hand, the ECG could have proven the pathophysiological effects of widespread ischemia of the entire anterior wall, the majority of the interventricular septum, and substantial portions of the antero-lateral wall, caused by significantly proximal LAD lesions. In N-STEMI patients with ST-segment elevation in the aVR lead, who also present significant ST-segment depression, trivascular disease or left main coronary artery disease has been observed [20]. In these cases, extensive subendocardial ischemia induced by the diastolic pressure of severe left ventricular dilatation and severe diastolic dysfunction has been proposed as the cause. Kosuge et al. [21] reported that proximal LAD lesion was found in patients with ST-segment elevation, as well as in patients without ST-segment changes or with ST-segment depression in aVR. However, only patients with ≥2 mm ST-segment elevation in >2 adjacent precordial leads and ≥1 mm ST-segment elevation in DI and aVL leads were included in the study, and it is possible that their selection favored the ECG pattern of the proximal LAD lesion.

In this study, the strongest association with proximal LAD lesions identified by coronary angiography was observed when there was electrocardiographic ST-segment elevation of ≥0.5 mm, particularly in lead aVL or lead aVR. Some studies have reported that a small subset of patients with LAD occlusion exhibited simultaneous ST-segment elevation in both the precordial leads and the inferior leads (DII, DIII, and aVF) [22]. Autopsy findings have revealed that, in most cases, the LAD artery wraps around the apex and gradually extends along the posterior interventricular wall.

Some studies have suggested that the number of cases with a sizable LAD that wraps around the apex is higher in patients with simultaneous anterior myocardial infarction with inferior ST-segment elevation. In cases with ST-segment depression noted in the inferior leads, the reverse situation was found, with a large LAD wrapping around the apex being found less frequently [23]. Sasaki et al. [23] demonstrated that the proximal lesion of a short LAD leads to ST-segment elevation in leads DI and aVL. However, the proximal lesion of a long LAD that extends around the apex was not related to ST-segment elevation in DI and aVL, or with reciprocal ST-segment depression in the inferior leads. The proximal LAD lesion concomitantly produces lesions in the two opposite electrical areas, the inferior and antero-lateral left ventricle wall. This kind of lesion could instead attenuate ST-segment elevation in the opposite antero-lateral leads and inferior leads, thus resulting in an isoelectric or even a depressed ST-segment in aVL, despite the existence of proximal LAD lesion.

The results from the present study showed that ST-segment elevation in the aVL lead was a more accurate indicator of proximal LAD lesion when patients with inferior ST-segment elevation were excluded from the analysis. The results are similar to those of other studies which suggest that aVL lead elevation does not necessarily indicate a proximal LAD lesion in cases where ST-segment elevation in all inferior leads is also present [24]. However, artery size should be considered as a variable when determining the occlusion site in the LAD.

To sum up, the location of the LAD lesion could be accurately determined by the 12-lead ECG in patients with acute anterior STEMI. ST-segment elevation of ≥0.5 mm in lead aVL, or any ST-segment elevation in lead aVR combined with ST-segment elevation in at least two contiguous precordial leads (including V2, V3, or V4), serves as an ECG marker with good sensitivity and both positive and negative predictive values for identifying a proximal lesion involving a middle or large branch of the LAD.

### 4.3. Anticipating the Location of the Lesion

The present work emphasizes that anterior AMI is an independent prognostic factor, as shown by multivariate analysis. The results are similar to previous studies conducted during both the fibrinolytic therapy era and the percutaneous coronary intervention era [25].

The results of the present study contradict the literature regarding the role of proximal LAD obstruction in the prognosis of AMI. In the present study, myocardial infarction installed as a consequence of proximal LAD obstruction was not associated with worse clinical outcomes, compared with patients with myocardial infarction produced by distal LAD occlusion regardless of the type of reperfusion therapy applied: percutaneous coronary intervention or fibrinolytic therapy. Karha et al. [26] showed that proximal LAD lesion lead to a higher death incidence during hospitalization in patients with anterior myocardial infarction compared to mid- or distal lesions. They used data from four different studies, comparing pharmacologic reperfusion therapies, and the results were obtained 90 min after fibrinolytic therapy. One possible explanation for such different outcomes between this work and others may be the difficulty in accurately identifying the proximal obstruction. Thus, all lesions after the second diagonal secondary branch were considered distal. Also, Karha and associates did not take into account the size of the lateral branch. Presumably, small lateral branches are not of major significance in terms of electrophysiologic or clinical importance. Therefore, according to the definition used in the mentioned study, the distal lesion of a first diagonal side branch could have been classified as a middle LAD lesion. This underscores the challenges faced in accurately determining the level of occlusion across individuals due to the considerable variability in coronary anatomy.

AMI consequent to proximal LAD lesion treated with PCI was associated with both higher short-term and long-term mortality, compared to distal LAD lesions, as suggested by Elsman and colleagues [6]. However, in the only research center in the mid-1990s, the use of antithrombolytic therapy was significantly different from that currently used. The time between onset of first symptoms and first balloon swelling was also not reported. In the present study, only patients with MI within the first hours of symptom onset were included.

Another explanation for the obtained results and literature data is the durability of reperfusion afforded by percutaneous coronary intervention, as well as contemporary aggressive therapy used in secondary prevention that neutralized the negative prognostic effect of the proximal LAD lesion observed in previous studies in patients who underwent primary percutaneous coronary intervention using older protocols.

Perhaps more surprising was the absence of differences in the clinical outcomes of fibrinolysis treatment of the proximal and distal lesions as defined by ECG. Consistent with the information provided by the present study, the CADILLAC study showed that 1-year survival was similar between patients with proximal vs. distal lesion (8.9 vs. 6.6%, *p* = 0.52) [7]. In the CADILLAC study, similar to the present work, the antithrombotic therapy was contemporary and efficiently conducted.

Therefore, it can be concluded that anterior myocardial infarction is an independent prognostic factor. Patients with anterior STEMI presented a higher mortality rate, as well as a higher rate of reinfarction and stroke, compared to the rest of patients. The prognostic relevance of the location of LAD lesion in the modern era of ST-segment elevation myocardial infarction treatment should be reevaluated.

## 5. Conclusions

The presence of ST-segment elevation in either aVL or aVR, associated with precordial ST-segment elevation in at least two contingent leads (including V2, V3, or V4), strongly suggests a proximal LAD lesion, often involving a medium or large diagonal branch, and offers meaningful predictive values. Conversely, ST-segment elevation in all inferior leads suggests an uncertain level of obstruction in patients with acute anterior STEMI. The ECG remains a critical tool in identifying the culprit artery in acute coronary events. While a proximal LAD lesion may not have a major prognostic impact in the modern era of STEMI treatment, the information provided by the ECG effectively assesses the target of PCI in the catheterization laboratory, especially in patients with patent infarct-related artery and severe stenosing lesions.

## Figures and Tables

**Table 1:** The culprit artery in patients treated with PPCI according to ECG patterns; eAMI, evolving myocardial infarction; cAMI, clinically established myocardial infarction; and PPCI, primary percutaneous coronary intervention.

Culprit Artery	eAMI-a *n* = 53	cAMI-a *n* = 48	eAMI-i *n* = 70	cAMI-i *n* = 18
LAD	50	45	1	1
LCX	1	-	12	1
RCA	-	1	50	15
Diagonal	1	1	2	-
Obtuse marginal	1	1	4	1
Left main	-	-	1	-

**Table 2:** ECG sensibility and specificity in identifying LAD occlusion, positive and negative predicting values. PPV, positive predictive value; NPV, negative predictive value; and CI, confidence interval.

ROC Area (AUC)	0.899			
Standard error	0.0239			
95% confidence interval	0.857–0.932			
z statistic s	16.683			
P statistical significance (Area = 0.5)	0.0001			
	PPV	95% CI	NPV	95% CI
	82.8	73.2–90.0	94.5	90.1–97.3
	Sensibility	95% CI	Specificity	95% CI
	87.80	78.7–94.0	91.98	87.1–95.4

**Table 3:** Diagnostic performance of ECG patterns in predicting proximal LAD lesions in anterior AMI: sensitivity, specificity, and predictive values (concomitant with ST-elevation ≥ 2 mm, maximal in leads V2–V4). ECG, electrocardiogram; PPV, positive predictive value; and NPV, negative predictive value.

ECG Pattern	Sensibility (%)	Specificity (%)	PPV (%)	NPV (%)
aVL+	82.1	55.4	78	61.1
aVR+	87	50	79.5	63.3
Proximal	92.5	44.1	83.8	65.2

**Table 4:** Sensitivity and specificity of the aVL + ECG pattern in predicting LAD occlusion (based on positive and negative predictive values). PPV, positive predictive value; NPV, negative predictive value; and CI, confidence interval.

ROC Area (AUC)	0.685			
Standard error	0.0494			
95% confidence interval	0.593–0.768			
z statistics	3.751			
p statistical significance (Area = 0.5)	0.0002			
	PPV	95% CI	NPV	95% CI
	78	67.5–87.7	61.1	43.5–76.8
	Sensibility	95% CI	Specificity	95% CI
	82.05	71.8–89.8	55	38.5–70.7

**Table 5:** Sensitivity and specificity of the aVR + ECG pattern in predicting LAD occlusiondpositive and negative predictive values. PPV, positive predictive value; NPV, negative predictive value; and CI, confidence interval.

ROC Area (AUC)	0.689			
Standard error	0.0498			
95% confidence interval	0.597–0.771			
z statistics	3.793			
*p* statistical significance (Area = 0.5)	0.0001			
	PPV	95% CI	NPV	95% CI
	79.5	69.6–87.4	63.3	43.9–80.0
	Sensibility	95% CI	Specificity	95% CI
	86.42	77.0–93.0	51.35	34.4–68.1

**Table 6:** Sensitivity and specificity of the proximal ECG pattern in predicting LAD occlusion positive and negative predictive values. PPV, positive predictive value; NPV, negative predictive value; and CI, confidence interval.

ROC Area (AUC)	0.683			
Standard error	0.0488			
95% confidence interval	0.599–0.758			
z statistics	3.745			
*p* statistical significance (Area = 0.5)	0.0002			
	PPV	95% CI	PPV	95% CI
	83.8	75.8–89.9	65.2	42.7–83.6
	Sensibility	95% CI	Specificity	95% CI
	85.7–96.7	44.12	27.2–62.1	85.7–96.7

**Table 7:** Mean time between symptom onset, ECG recording, and initiation of fibrinolytic treatment or PCI. F time, fibrinolysis time; PCI, percutaneous coronary intervention.

	Mean Time (min)	Standard Deviation	95% CI	
Time from symptoms onset to ECG recording (E time)	141	52.76	136–147	
Time from symptoms onset (E time) to PCI or fibrinolysis (PCI time/F time)	215	58.42	208–220	
Time from ECG recording (E time) to PCI or fibrinolysis (PCI time/F time)	73	36.18	70–77	
Time from ECG recording (E time) to fibrinolysis (F time)	63	33.66	58–67	*p* < 0.0001
Time from ECG recording (E time) to PCI (PCI time)	84	35.63	78–89	

**Table 8:** Final end-points in patients with proximal LAD occlusion (comparison between PCI and FT) and patients with distal LA occlusion. PCI, percutaneous coronary intervention; FT, fibrinolysis time.

	LAD Proximal Occlusion	LAD Distal Occlusion	*p* Value	LAD Proximal Occlusion
Mortality	12%	17%	0.3832	
Reinfarction	23%	26%	0.7129	
Stroke	7%	9%	0.7604	
Composite end-point	PCI	FT	-	0.6862
	21%	18%		

## Data Availability

All data are available in the archive (database) of the Clinical County Emergency Hospital of Oradea, Oradea, Bihor County, Romania.
